# Vascularity and Dynamic Contrast-Enhanced Breast Magnetic Resonance Imaging

**DOI:** 10.3389/fradi.2021.735567

**Published:** 2021-12-09

**Authors:** David E. Frankhouser, Eric Dietze, Ashish Mahabal, Victoria L. Seewaldt

**Affiliations:** ^1^Department of Population Sciences, City of Hope National Medical Center, Duarte, CA, United States; ^2^Department of Astronomy, Division of Physics, Mathematics, and Astronomy, California Institute of Technology (Caltech), Pasadena, CA, United States

**Keywords:** breast cancer, blood vessel, early detection, MRI, radiomics, artificial intelligence, vascularization

## Abstract

Angiogenesis is a key step in the initiation and progression of an invasive breast cancer. High microvessel density by morphological characterization predicts metastasis and poor survival in women with invasive breast cancers. However, morphologic characterization is subject to variability and only can evaluate a limited portion of an invasive breast cancer. Consequently, breast Magnetic Resonance Imaging (MRI) is currently being evaluated to assess vascularity. Recently, through the new field of radiomics, dynamic contrast enhanced (DCE)-MRI is being used to evaluate vascular density, vascular morphology, and detection of aggressive breast cancer biology. While DCE-MRI is a highly sensitive tool, there are specific features that limit computational evaluation of blood vessels. These include (1) DCE-MRI evaluates gadolinium contrast and does not directly evaluate biology, (2) the resolution of DCE-MRI is insufficient for imaging small blood vessels, and (3) DCE-MRI images are very difficult to co-register. Here we review computational approaches for detection and analysis of blood vessels in DCE-MRI images and present some of the strategies we have developed for co-registry of DCE-MRI images and early detection of vascularization.

## Introduction

### Vascularization and Breast Cancer Initiation

Angiogenesis is a dynamic process and an important early step during breast cancer initiation and progression (1, 2). The morphologic quantitation and characterization of blood vessels in a biopsy specimen relies on indirect measures such as microvessel density (number of small and tortuous vessels, immunohistochemistry (factor VIII, CD31, CD34)) (2–6). High microvessel density predicts metastasis and poor survival in women with breast cancer, including women with early-stage breast cancer (Stage 1) (7–16). While morphologic characterization of vascularity has strong prognostic value, there is variability (1) between different pathologists—there is significant inter- and intra-reader variability and (2) within a tumor—frequently a single tissue slice does not adequately capture microvessel density of an entire tumor. The variability of morphological characterization of blood vessels becomes particularly limiting when evaluating (1) response to neo-adjuvant chemotherapy and (2) women who are at high-risk for developing a breast cancer (e.g., germline *BRCA* mutation). Consequently, imaging strategies are being developed and optimized for early detection of vascularization, and ultimately, neovascularization.

### Breast DCE-MRI

Dynamic contrast-enhanced magnetic resonance imaging (DCE-MRI) is routinely used to screen women at high-risk for breast cancer, evaluate the extent of local invasive breast cancer, and assist in planning of neo-adjuvant and adjuvant therapy (17). DCE-MRI utilizes timed pre- and post-contrast imaging; consequently, the semi-quantitative dynamics of contrast uptake and intensity (initial peak enhancements, delayed phase-washout) are routinely used for radiologic evaluation of DCE-MRI (18). DCE-MRI has proven to be highly sensitive and has revolutionized early detection of breast cancers, particularly in women who are at increased risk for breast cancer (19–31). Consequently, breast DCE-MRI has become standard of care and made it possible to obtain a high-resolution image of breast cancers and their associated vasculature. Despite the clinical success of breast DCE-MRI to image the breast with high sensitivity and resolution, the ability of DCE-MRI to image blood vessels with high precision remains a work in progress. Recently, through the new field of radiomics, contrast dynamics are being used to evaluate vascular density, vascular morphology, and detection of aggressive breast cancer biology. In addition to directly detecting and quantifying the vascularity, the indirect effects of altered vascularity such as perfusion and blood oxygen levels can be measured using both DCE-MRI alone and DCE-MRI in combination with other imaging modalities. These indirect approaches detect increased blood flow and hypoxia and have been shown to be diagnostic of breast cancer and predictive of treatment response (32–38). However, this review will focus on efforts to detect vessels in DCE-MRI of the breast.

### Importance and Limitations of MRI-Based Vessel Detection in Breast Tissue and Cancer

DCE-MRI is standard-of-care for high-risk cancer screening and surgical staging and is not readily replaced. Furthermore, DCE-MRI time-sequence imaging data provides an opportunity to perform kinetic and pharmacokinetic modeling; this modeling holds promise to better characterize tumors (28, 30, 39, 40), monitor treatment effects (25, 31, 41), and plan radiation and surgical interventions (29, 42–44). Given the importance of DCE-MRI imaging, recent efforts have focused on detecting blood vessels in DCE-MRI images. However, the detection of blood vessels, particularly new blood vessels, in DCE-MRI images presents significant challenges.

First challenge is that DCE-MRI is not able to directly image biology. The presence of vessels is detected, both manually and computationally, based on high contrast enhancement and their linear morphology (shape). The fact that DCE-MRI provides dynamic 3D images of all tissue is simultaneously both the reason why it has so much potential benefit (wide-scale adoption and utility) and the reason why vessel detection is so difficult.

Second challenge is the resolution of MRI. Although MRI equipment with strong magnetic fields (>7.0 Tesla) can achieve a resolution <200 μm, the standard clinical MRI equipment used for breast cancer screening employs significantly weaker magnetic fields (1.5 or 3.0 Tesla) (45). This means that most MRI can only resolve features between 0.5 and 1.0 mm in size (46); small blood vessels range in size between 4 μm to < 1 mm (47). Consequently, DCE-MRI lacks the resolution to detect small or micro-vessels (48, 49).

A third issue is that breast tissue is inherently difficult to image. Unlike other tissue, where blood vessel detection has high accuracy, breast tissue presents many unique challenges (50–54). The human breast is highly heterogeneous both within the breast of individuals women and between women. Human breasts vary in size, the ratio of glandular tissue to adipose tissue, and the amount of collagen/extracellular matrix; furthermore, the human breast is subject to hormonal fluctuations during the menstrual cycle and undergoes complex changes during a woman's lifetime (puberty, pregnancy, lactation, involution, and menopause). The heterogeneity of the human breasts requires that imaging algorithms must be sufficiently flexible to have general applicability. Since breast tissue spans a range of densities and compositions, contrast enhancement changes within the breast and the intensity between background and tissue are not constant (48, 55). The breast also includes both adipose and fibroglandular tissues and both can appear on DCE-MRI images to have linear morphology (49, 55). Since blood vessels are detected based on contrast enhancement and morphology, accurately detecting blood vessels in the breast is very difficult. Additionally, the breast is highly deformable so movement during scans can change both the position and the shape of the breast resulting in imaging artifacts and making blood vessel detection even more difficult (49).

While it is challenging to perform vascular analysis of DCE-MRI (particularly in 3D), DCE-MRI is standard-of-care for breast cancer screening, surgical planning, and evaluation of response to neo-adjuvant chemotherapy. The advantages of DCE-MRI as a sensitive, non-invasive method to monitor for and detect lesions have made it a routine clinical procedure (19–21). Given the importance of DCE-MRI in clinical care, it is unlikely that it will soon or easily be replaced as the clinical standard-of-care by another imaging modality. Therefore, it is important to develop effective strategies to detect blood vessel density and morphology in DCE-MRI images. In this article, we aim to review computational approaches for detection and analysis of blood vessels in DCE-MRI images and present some of the strategies we have developed for co-registry of DCE-MRI images and detection of blood vessels and neovascularization.

## Computational Approaches in Breast DCE-MRI

Computational analysis of breast MRI images is an active area of research that involves a wide range of methods and research goals. Here, we will provide a brief review of the non-vessel computational approaches used to analyse breast MRI images. This review will serve to introduce the wide range of computational and mathematical modeling approaches used for different breast cancer research. These non-vessel approaches will be used to inform and contrast with the derth of research in vessel detection. In this section, we will separate our review topics into (1) tumor detection and characterization or (2) radiomic analysis. The goal of radiomic analysis can either be detection or characterization of lesions/tumors, the specific goal of the analysis drives which approach is selected. Tumor Detection and Characterization is performed by applying mathematical and computational analysis to the imaging data. Radiomics first extracts quantitative features from the imaging data, then organizes the features in a database, and finally uses mathematical and computational approaches to perform analysis on the database (56). Here we will briefly survey and compare the computational approaches used for (1) Tumor Detection and Characterization and (2) radiomics.

### Tumor Detection and Characterization

Tumor Detection and Characteristics analysis is conducted both for research and to improve clinical decision making. Analysis uses morphologic features, enhancement kinetics, or a combination of both. To support clinical decision making, clinical aided systems work with human experts to improve both (1) early identification of tumors and (2) the sensitivity/specificity of breast imaging (57, 58). Many clinical aided systems have been developed and commercialized for mammography. In contrast, development of clinical aided systems for breast DCE-MRI is in its nascent stage and the systems that have been developed have not been widely tested outside the data set for which they were developed (58, 59). This lag in the development of clinical aided systems for breast MRI is primarily the result of lack of standardization of DCE-MRI across centres and institutions. Key differences in DCE-MRI protocols include the (1) specific gadolinium contrast agent used, (2) amount of contrast given, (3) magnet strength, and (4) image acquisition strategy. Despite these challenges, approaches have been developed and, within their data set, demonstrate excellent sensitivity and specificity.

The first type of Tumor Detection and Classification methods analyse the dynamics of the contrast agent. To identify tissue with malignant potential, the earliest methods of this type used kinetic curves, which show changes in contrast enhancement over time. The kinetic curve was constructed by plotting the signal intensity over time and classifying the curve based on the rate of contrast uptake and then washout. The shape of the washout curve was used to diagnose the tissue; benign or indeterminate tissue has slow uptake or plateauing intensity over time; malignant tissue has both rapid uptake and washout (60–66). The rapid uptake/washout of malignant tissue is attributed to the increased vascularity associated with tumors (66–68). These models, however, are often considered semi-quantitative and, due to inconsistencies between DCE-MRI protocols and machines, need to be tuned and adapted for each new study (59, 69). A more quantitative approach is to use pharmacokinetic models that apply mathematical equations to model the flow of contrast agent through the vasculature into the tissue (70); the most common model is a variant of the Tofts model (70–75). These flow models allow for measurement and comparison of physiologically relevant parameters; parameters measuring volume of contrast transferred (*K*^*trans*^) and vascular volume (*v*_*p*_) are able to distinguish between malignant and benign tumors (48, 69). The parameters *K*^*trans*^ and *v*_*p*_, similar to kinetic curves measuring contrast uptake/washout, are assumed to correlate with the increased vascularity of malignant tissue. When compared to classifying kinetic curves, pharmacokinetic models have also been shown to out-perform kinetic curves (69, 76) and reduce the variability between DCE-MRI protocol and noisy signal (77). These models, however, also make assumptions about contrast flow and require multiple post-contrast time points; these assumptions limit their general applicability (74, 77, 78).

The second type of Tumor Detection and Classification methods involve machine learning. Machine learning approaches have been employed frequently and span the machine learning spectrum from clustering to deep learning neural networks. A number of reviews about machine learning in breast DCE-MRI focus on either the types of methods (79), the goal of the method (58, 80), or the conclusions of the study (57, 81). Starting with the machine learning approaches, we will provide a concise, non-exhaustive survey of the more popular machine learning methods being used and briefly describe each approach.

Fuzzy C-means clustering is one of the most widely used unsupervised approaches to analyse breast DCE-MRI images. The general strategy of Fuzzy-C means clustering algorithms is to classify input data into groups based on algorithms that minimizes in-group variability. Although many improvements have been made to the algorithm (82–86), Fuzzy C-means analysis of breast DCE-MRI images has been primarily limited to segmenting the boundaries of a lesion (84).

Machine learning algorithms that use a supervised training approach include logistic regression, linear discriminant analysis, random forests, and support vector machines. All these approaches require “training” on a fully classified set of data (i.e., DCE-MRI with all lesions labeled as either malignant or benign). After a training set is analyzed, each of these approaches can then be applied to a second labeled set (independent of the training) to test sensitivity and specificity. Although they share a training approach, these methods have important differences in how they perform classification. Logistic regression (for two classes) and linear discriminant analysis (for more than two classes) are both linear methods. Support vector machines and random forests (for both two and multi-class scenarios) are more robust non-linear methods. All these supervised machine learning methods have been used to classify malignant tissue (87–92).

The third type approaches used in Tumor Detection and Classification include artificial neural networks and deep learning (i.e., neural networks with multiple hidden layers). Neural network architecture contains an input layer, at least one hidden layer, and an output layer. Deep neural networks (i.e, deep learning methods) perform both feature extraction and classification. In the training phase, deep learning methods “learn” what features best classify the input data (81, 93). Because it is well suited for image recognition, the most common neural network architecture for DCE-MRI is convolutional neural networks (CNNs). The convolutional layers of a CNN extract imaging features while maintaining spatial relationships between features. The discovered features are aggregated by pooling layers before the output layers generate a classification. In addition to being applicable to Tumor Detection Classification (94, 95), the flexibility of CNNs address a wide range of breast DCE-MRI related research issues including fibroglandular tissue segmentation (96, 97); breast segmentation (95, 98); and detection of lymph node metastasis (99). The disadvantage to deep learning is that it requires a large, annotated training data set. However, transfer learning, where a network is pretrained on a large imaging database, has shown promise for reducing the required size of training data sets (80, 95–97). In other applications, deep learning has shown to out-perform standard computer vision and experts (94, 100); however, in MRI, deep learning approaches have not yet out-performed experts (58).

### Radiomics

Radiomics is a field of research that views medical images as quantitative data; this data represents the phenotypic, genotypic, and molecular characteristics of the tissue. The goal of radiomic studies is to associate the quantitative features extracted from images to both qualitative (e.g., clinical variables and outcomes) and quantitative information (e.g., biomarkers, gene expression, or other relevant -omic measurements). Radiomic approaches have many research applications including diagnostic tools, clinical decision support, and hypothesis testing and generation (56, 101, 102). For comprehensive summary of radiomics in breast cancer, many researchers review radiomics studies based on their research goals (19, 56, 101–106). To achieve these research goals, radiomics has employed wide range of computational approaches. The computational approaches can be categorized based on the three different parts of the radiomic analysis: features extraction, feature selection, and model building (56). The goal of features selection and model building are, respectively, to classify features and then build predictive models so that unlabelled images can be classified. The main difference are the variables that are classified: in addition to detecting benign vs. malignant tissue, radiomic studies classify continuous variables to define cancer subtype, diagnosis, and prognosis. Since features selection and model building primarily use machine learning methods similar to those described in the machine learning section of this section, we will limit our review of computational approaches to those used in feature extraction.

Feature extraction is the process of identifying features from an image. The features can be any quantifiable data produced from either the full image or a segmented region of interest within the image. The main categories include morphology-, histogram-, texture-, and transform-based features (56). This process is not unique to radiomics and can include some already discussed features such as contrast kinetic features (i.e., washout dynamics and pharmacokinetic parameters). However, it is typically distinct from deep learning in that the extracted features are selected manually and not discovered by an algorithm (57).

The types of features extracted fall into two categories: semantic and agnostic (101). Sematic feature extraction attempts to capture prognostically meaningful characteristics such as the BI-RADS defined features of a lesion (e.g., density, spiculation, and vascularity). These features, which are visually identifiable by radiologists, primarily fall into the morphology- and histogram-based categories. Agnostic features are not directly observable and have to be computed or extracted from the image. The features most unique to radiomics are the texture- and transform-based features. The gray-level co-occurrence matrix (GLCM) is a texture-based feature used to represent tumor heterogeneity. GLCM is calculated by determining the frequency of neighboring pixels with specific intensities (106, 107). This texture-based analysis captures the spatial distribution of intensity. GCLMs and their second-order features have had success identifying lesions (87, 90, 108), characterizing molecular and histopathologic subtype (109–113), and predicting prognosis and therapy response (114–118). Transform-based features are features that are extracted after applying a function to the image to generate a new image. Examples of transformations include Fourier, Wavelet, and Laplacian of Gaussian transforms. Instead of the spatial dimension, these transformations convert the image to investigate texture in an alternative dimension such as time or frequency. Although not as widely used as GLCM, transform-based approaches in breast cancer have also identified, characterized, and predicted response (119–123).

The wide-spread adoption of radiomics has been hindered by both the (1) large amount of data needed for training and (2) limitations of DCE-MRI (e.g., contrast type and administration protocols, magnetic strength, etc.) (19, 57, 104). Combining radiomics with deep learning has shown promise for improved accuracy. Transfer learning, in particular, may be a type of deep learning that can reduce the size of data sets and time required for training.

## Automated Vessel Detection

Vessel detection represents a small subset of breast MRI research (Table 1). It has so far been used to either: (1) compare the gross characteristics of the vascular network between ipsilateral tumor bearing breast and the contralateral tumor free breast; or (2) improve the detection specificity of tumorous vs. benign lesions. In the context of breast cancer research, vessel detection in breast MRI first used manual detection of vessels by experts. Expert detection was typically performed on two-dimensional (2D) projections of the three-dimensional (3D) volumetric image. These 2D projections were also used for the first fully computational detection algorithms. Methods for full 3D computational detection of vessels in MRI were first reported in the late 1990's. However, 3D vessel detection in research has only recently been reported. The limited number of studies using 3D vessel detection are likely due to the considerable challenges of vessel detection in breast DCE-MRI.

**Table 1:** Vessel detection studies in breast MRI.

**References**	**Number of dimensions** **(2D or 3D)**	**Vessel detection method**	**Findings**
Lin et al. (49)	2D	Hessian morphology filter	Removing pixels belonging to large vessels reduced false positives when identifying suspicious lesions
Fusco et al. (124)	2D	Hessian morphology filter	Ipsilateral increase in vascularity was associated with malignancy; vessel detection performance: 79% true positive, 20% false positive, and 2% false negative rates
Petrillo et al. (125)	2D	Hessian morphology filter	Tumor location in the breast correlated with feeding vessel location; vessel detection demonstrated good agreement with expert assessment
Wu et al. (126)	2D	Hessian morphology filter	Vessel voxels and volume are reduced in neoadjuvant treatment responders
Kostopoulos et al. (127)	2D	Seed growth	Multiple texture-based parameters of vessels show statistically significant differences between breasts with benign vs. malignant tumors
Gierlinger et al. (128)	3D	Seed growth	The algorithm detected similar vessel structure from two different scans collected from the same individual
Vignati et al. (129)	3D	Hessian morphology filter	Vessel detection algorithm shown to have 89% detection rate with 98% sensitivity
Vignati et al. (130)	3D	Hessian morphology filter	Removing vessel structures from images decreased false positive rate of parenchymal lesions by 68%
Vignati et al. (131)	3D	Hessian morphology filter	Vessel volume was decreased in responders vs. non-responders of neoadjuvant chemotherapy
Kahala et al. (55)	3D	Hessian morphology filter	A vessel detection algorithm with center line tracking to fill-in incomplete vessels demonstrated 86% sensitivity and 88% specificity
Wu et al. (48)	3D	Hessian morphology filter	Malignant lesions have a greater number of lesion-associated vessels

### Computational Challenges of Vessel Detection in DCE-MRI

It is difficult to automatically detect blood vessels in a non-specific imaging method such as DCE-MRI. DCE-MRI does not directly measure biology; therefore, the characteristics of the breast tissue increase the computational difficulty of measuring blood vessels. Initial efforts relied on experts who manually identified, measured, and scored blood vessels in DCE-MRI images. Since MRI provides no inherent method to distinguish vessels, detection is performed based on both their high enhancement in MRI due to rapid contrast uptake and their linear, network-like appearance (i.e., morphology) (49, 129, 132). Robust detection of wide range of vessels sizes requires image analysis at multiple scales (or resolutions). Some computational algorithms require reprocessing of an image at different scales, since a linear structure, if magnified sufficiently, no longer will appear linear. The number of and increment between scales depends on the (1) sensitivity of the algorithm and (2) MRI resolution. Finally, all contrast enhancement in DCE-MRI is reported as a numerical intensity; therefore, vessels can escape detection if: (1) nearby tissues exhibit rapid contrast uptake which occludes vessels; or (2) sufficiently small vessels do not contain enough contrast agent to achieve sufficient enhancement (49, 55, 133). Both these scenarios result in gaps appearing in the vascular network. These missing data require computational strategies to both detect and fill gaps when constructing a complete and accurate vascular network.

A robust computational algorithm must detect blood vessels with (1) similar accuracy between patients and (2) ideally, be independent of the MRI machine and protocol used. Differences in MRI machines can include different resolution (resulting from different magnetic field strengths), ranges of enhancement intensity, and software versions. Differences between breast DCE-MRI contrast administration protocols can result in contrast enhancement characteristics that cannot be directly compared. These include differences in the type of contrast agent, changes in contrast agent dose or administration rate, and different acquisition protocols or number of scans. Some of these factors can be minimized or corrected through analysis methods and image processing. For example, pharmacokinetic modeling avoids comparing intensities directly by instead comparing the model parameters after fitting. Intensity functions can be applied to the image before analysis to normalize the range of intensity within a DCE-MRI image (48). The intensity transfer function will enhance the low intensity tissue while suppressing the image background. Intensity thresholds can also be applied to remove background noise but generally require calibration within each image so that low intensity features are not lost (134).

Finally, a computational challenge that is specific to the breast is image co-registration. Although co-registration is not required for detecting vascularity in a single DCE-MRI image, co-registration is required to detect changes in vascularity (or other imaging feature) over time. Breast registration is difficult because the breast is highly deformable which results in a complex combination of both affine and non-rigid transformations (135–138). Additionally, many times the breast itself has changed over time either because of either natural biological cycles or malignant transformation. Together these make registration of serial breast MRI images exceptionally challenging.

### Computational Blood Vessel Detection in Breast MRI to Date

#### 2-Dimensional Algorithms

There are relatively few computational methods used to detect blood vessels. The majority of computational methods to detect blood vessels in breast MRI have been conducted in 2-dimensional (2D) projections of MRI images (Table 1). The most useful type of projection for detecting blood vessels is called the maximum intensity projection (MIP). This is an image that takes only the pixel with maximum intensity along one dimension of the 3D volume to create a 2D projection (111, 139). The resulting image highlights the features that exhibit the highest contrast enhancement. Since the contrast agent is transported through vessels to the tissue, the difference in enhancement between the pre-contrast scan and the first post-contrast scan (subtraction image) is typically used as the starting point to identify the vessels. In addition to highlighting the high intensity blood vessels in the image, the MIP image also reduces background noise which makes vessel detection easier.

Vessel detection strategies in MIP images primarily involve the use of a morphology-based filter (Hessian-filter) that selects linear or filamentary structures within the image (for a full discussion of Hessian-filter method see the 3D detection section). A number of studies have used MIP-based detection to analyze blood vessel characteristics and proximity to malignant breast tumors (49, 124–126).

A second computational method for blood vessel detection uses a technique called regional seed growth. A seed growth algorithm starts with seeded locations around an image and expands the seeds to contingent pixels (140). The seed eventually grows to include all similar adjacent pixels in the image. Although not specific to vessel detection, this method would appear well-suited to detecting the high intensity vascular network in breast DCE-MRI. However, in breast DCE-MRI images, the breast fibroglandular and adipose tissue (non-vascular tissue) can be similar in appearance to blood vessels. This similarity may explain why the regional seed growth is not frequently used to analyze breast MRI images. Kostopoulus et al. reported vessel detection for MIP images that used seed growing algorithm (127). This study (1) calculated quantitative metrics of both the vascular network and the non-vascular portion of the breast and (2) compared these characteristics in breast MRI images with malignant lesions vs. benign lesions vs. no lesions. Although this study compared a number of metrics calculated for the detected vessels, only the standard deviation of intensity in pixels identified as vessels showed a significant difference between malignant, benign, and normal breasts. They suggested this was due to variation in pixel intensity near the malignant tumors.

Although 2D vessel detection is less difficult conduct than in 3D, 2D detection has drawbacks. First, the 2D projection loses spatial information in the direction of the projection. Therefore, it is not possible to measure either the (1) distance between vessels and other imaging features (e.g., tumor) and (2) 3D characteristics of the vessels (i.e., curvature, torsion, tortuosity, and volume). Second, enhancing tissue can completely occlude or obscure vessels. Despite these limitations, MIPs remain a useful visualization tool and have been used as a guide to improve machine learning detection of malignant lesions (141).

#### 3-Dimensional Algorithms

To date, there have only been a few studies that have reported computational detection of vessels in 3D breast MRI (48, 55, 130). One modification of a seed growth algorithm has recently been reported and applied to a single test case (128), but all other 3D vessel detection algorithms are based around calculating the Hessian to construct a morphologic filter (Table 1).

**Figure 1:**
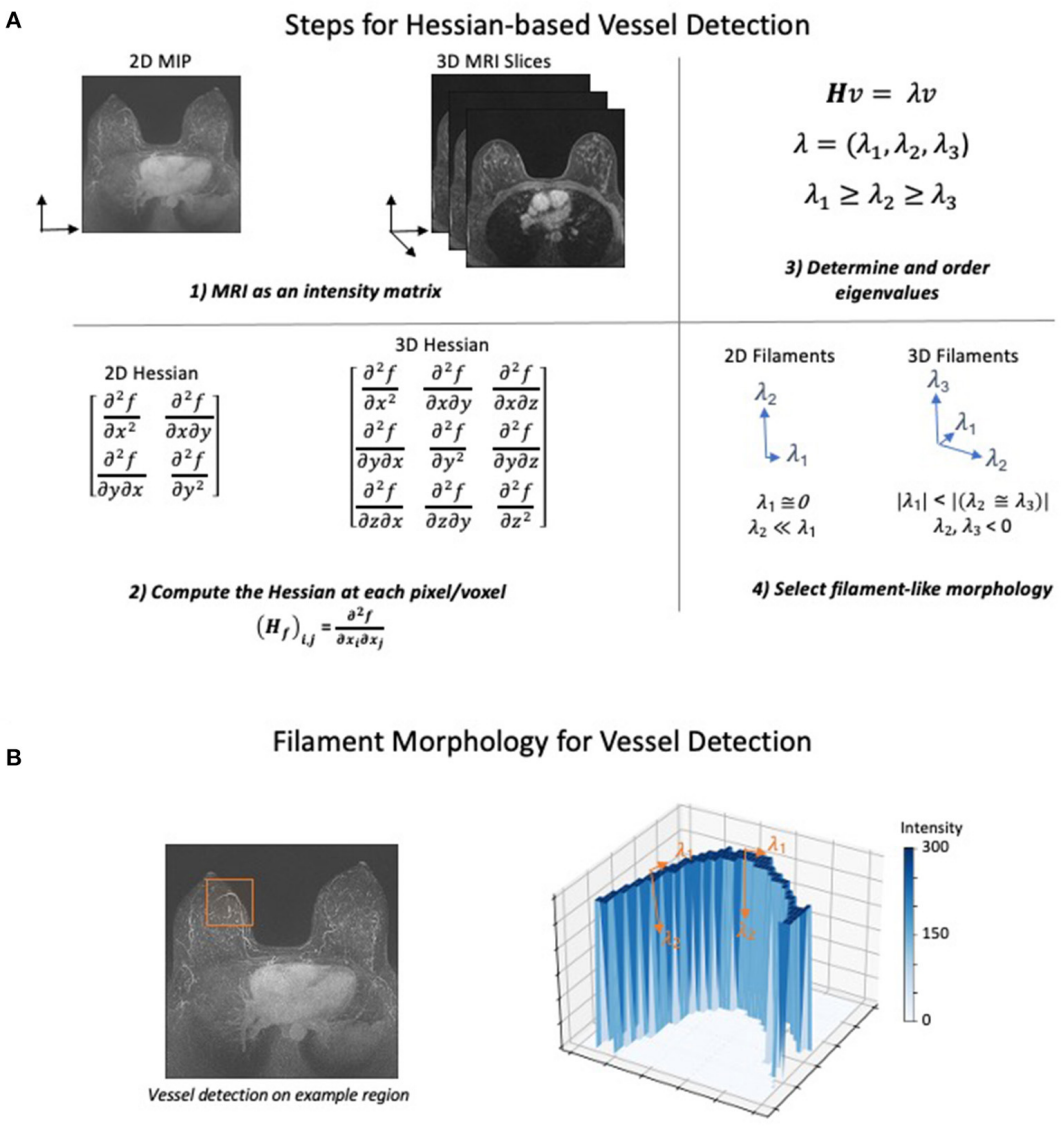
Hessian-based Algorithm for Vessel Detection. The general steps for detecting filaments using a Hessian matrix-based approach. **(A)** (1) The MRI is converted to either a 2- or 3-dimensional matrix where the intensity of each pixel is recorded as a numerical value. (2) Next, using the number of dimensions in the image, the Hessian matrix is computed at each point of the intensity matrix. The Hessian matrix contains the second derivative of intensity with respect to each spatial dimension. (3) Eigenvalues of the Hessian are computed and ordered based on their value to prepare for morphology selection. (4) Ordered eigenvalues can be filtered to determine whether a pixel has filament-like morphology. The selection for filaments in both 2D and 3D attempt to identify pixels where the second derivative of intensity is low, and the second derivative of all other dimensions is large and negative. Other morphologies such as spheres and sheets can also be identified using different selection criteria. **(B)** To demonstrate how the eigenvalue criteria identify filament-like structure, a vessel containing subregion of a MIP image was selected (left). The intensity in the selected 2D region was plotted as a surface after background normalization and an intensity transform function was applied (right). Forming a sharp ridge, the high values of intensity correspond to a vessel. The eigenvalues of two points are annotated to describe how the filament selection criteria identify vessel-like structures. Low rates of intensity change occur in the direction of the vessel (λ_1_) while large decreased in the rate of intensity change occur in the orthogonal direction (λ_2_).

The Hessian was first applied to imaging data to detect vessels by Frangi et al. in 1998 (132). The Hessian approach uses a simple series of steps that ultimately select for different morphological structures present in the image (Figure 1A). First, the MRI is converted to a matrix of intensity values. Second, the Hessian is calculated on the intensity at each pixel or voxel in the image. The Hessian is a matrix of second derivatives which determines the change in the rate of change (acceleration) of the intensity in all directions. Third, eigenvalues are used to determine the directions in which the second derivatives of intensity are the largest. Finally, a morphologic filter is applied which allows selection of either sphere-, sheet-, or filament-like structures in the image. Conceptually, this filter identifies intensity features where the rate of change in intensity is small in one dimension and large and negative in all others (Figure 1B). Most methods additionally apply both preprocessing to normalize or filter intensities and postprocessing to remove small unconnected lines (noise) and fill in gaps in the vascular network.

The Hessian approach has been applied to breast DCE-MRI to detect vessels with some success. Vignati et al. detected the vascularity so that the vasculature could be removed thereby improving tumor detection (130). In a later study, Vignati et al. showed a difference in vessel count and vessel volume in responders vs. non-responders after neoadjuvant chemotherapy (131). Kahala et al. developed a vessel detection algorithm with a sensitivity and specificity of 86 and 88%, respectively (vs. vascular networks detected by radiologists) (55). The most comprehensive study was reported by Wu et al. (48). This study used detected vascular networks to compare vessel characteristics and pharmacokinetic parameters between benign and malignant tumors. The vessel count and two blood flow parameters (volume transfer coefficient and plasma volume fraction) from their models showed a significant increase in malignant tumors (relative to benign lesions). The observed differences were used to develop a multivariate logistic regression model to improve malignant tumor detection.

**Figure 2:**
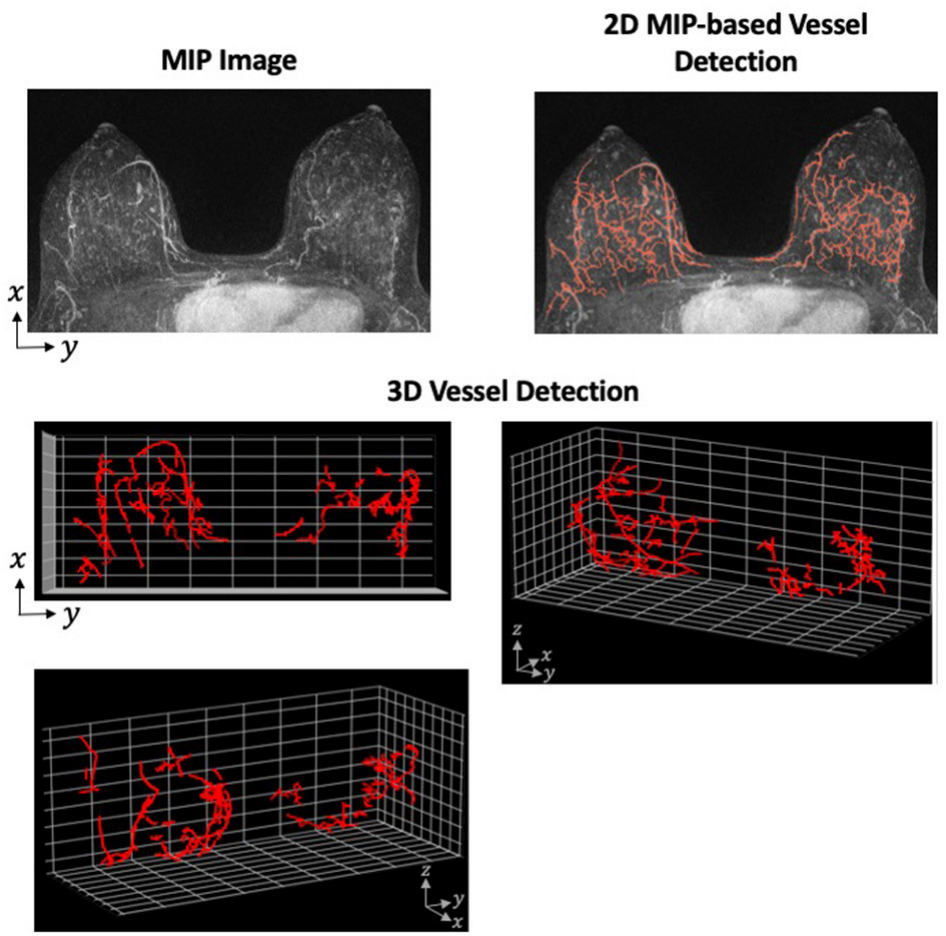
Vessel Detection Performed in 2- and 3-Dimensions. A Hessian-based approach was applied to both 2D MIP and full 3D MRI using a single sample from our data set. The vessel detection algorithm was based on a combination of methods described by Vignati et al. (130) and Wu et al. (48) with parameters determined using our data set. The MIP images shows many filamentary-like enhancing structures at the base of the breast. These structures were consequently detected as vessels in the 2D approach. However, the distance between these structures on the z-axis are not preserved when projected to 2-dimensions. When detecting vessels using the full 3D image, these enhancing structures prove to be unconnected and are, therefore, not detected as vessels.

Although the application of the Hessian-based morphologic filter is straight-forward, the difficulties in this method arise when tuning the preprocessing, postprocessing, and morphology selection parameters. These parameters require careful tuning so that the algorithm is robust within a particular data set. Additionally, an algorithm developed and successful detecting vessels on one data set is not necessarily transferable to another data set with a different MRI machine or protocol. However, the advantages of 3D vessel detection outweigh the reduced difficulty of 2D. One of the primary disadvantages of 2D detection is the loss of spatial relationship in the direction of the projected dimension. For an example of how the projection of 3D structure on to 2-dimensions can cause unconnected fibroglandular tissue to appear more like vessels, see Figure 2.

## Discussion

The promise of MRI is as a non-invasive, quantitative assessment of an individual's tumor. The goal is to be able to make personalized clinical decisions based on the information obtained from MRI. This is demonstrated by the large field of existing research which uses breast MRI to characterize breast cancer, identify cancer subtype, and predict both prognosis and treatment response. However, detection and assessment of the vessels is seldom performed in this large body of research. Despite increased vascularity causing the contrast dynamics that allow tumor detection, detection of the vascularity and subsequent analysis remains sparse (66–68). In comparison with MRI research in general, the scope of research involving vessel detection from breast MRI is less broad: it shows increased vascularity to be associated with tumors and invasion (142–146). Given the important role vessels play in nearly all aspects of breast tumors, the lack of research attention to detect vessels in MRI is likely due to the challenges presented and not a lack of interest.

Breast MRI presents significant challenges for any quantitative assessment. Although these challenges are not unique to vessel detection, many of these challenges (e.g., fibroglandular tissue, small size of vessels, etc.) make vessel detection particularly challenging. However, MRI is data-rich and has proved to contain information that can be translated into clinical diagnosis, prognosis, and therapeutic assessment. We think the information encoded in the vessels will further improve the clinical impact of breast MRI in the future.

## Future Directions

Currently, blood vessel detection in DCE-MRI lags far behind other research and clinical application of other computational analysis. This is not only because of the challenges presented by MRI-based detection and breast tissue but also because the utility of vessel detection has largely been limited to aid lesion detection and characterization. In the short term, research involving vessel detection in MRI can progress on two fronts: improved reproducibility and more clinically relevant tumor characterization and prognosis. Longer term, more advanced vessel detection methods from computer science can both advance the field and lower the barrier for applying vessel detection algorithms to clinical research.

Although current vessel detection algorithms have shown relatively high sensitivity and specificity within a single data set, no vessel detection algorithm has been compared between data sets. To become more widely adopted for research and, eventually, clinical purposes, vessel detection algorithms must be shown to be robust and reproducible. This will require developed algorithms to be performed in and compared between studies that use different MRI machines and different MRI protocols. While challenging, a robust and reproducible blood vessel detection algorithm would provide important clinical and scientific information. The solution to many of these technical challenges fall within the domain of computer science and computer vision research which can be either developed or adopted to MRI-based research. The development of deep learning algorithms could also reduce the difficulty of implementing vessel detection in the research setting. Currently, vessel detection algorithms are both custom built for the dataset and time intensive. Previously reported vessel detection studies can provide a framework for developing new algorithms, but because of differences in imaging parameters and machines, parameter tuning and optimizing the algorithm are still required. These steps are challenging and must be repeated when applying any developed algorithm to new datasets. Designing vessel detection algorithms that are more transferable between datasets would reduce the barriers to research applications. Transfer learning paired with an optimization procedure that uses a small subset of images from a new dataset could be one solution to algorithms that are more widely and easily applicable.

Toward the application of vessel detection for research purposes in breast MRI, we are currently developing a vessel detection algorithm to associate vascular characteristics with aggressive tumor growth. Using a breast screening database that contains examples of both aggressive and slow-growing tumors, we plan to assess both global and tumor-associated vessel characteristics. To determine whether changes in the vasculature can be associated with aggressive tumors, we plan to compare those characteristics between the aggressive and slow-growing tumors. The characteristics will be derived from both hypothesis-driven quantitative metrics and machine-learning approaches to extract features from the vessel-segmented images. We view this as a radiomic-like approach to vessel analysis which has not previously been attempted. To build this capability, we have developed both 2D MIP-based and 3D vessel detection algorithms by adapting previous approaches to our data set (Figure 2). Using radiomic and deep learning approaches, our goal is to identify texture-based features of the vessels to help identify biologically aggressive from slow-growing tumors.

## Author Contributions

DF wrote much of the manuscript. ED edited the manuscript. AM contributed ideas and edited the manuscript. VS contributed ideas and edited and revised the manuscript. All authors contributed to the article and approved the submitted version.

## Funding

Funding sources include National Institutes of Health/National Cancer Institute (NIH/NCI) grants R01CA170851, P20CA24619, R01CA192914, and U01CA189283 (all to VS) and P30CA033572 and Welcome Leap. The funders had no role in study design, data collection and analysis, decision to publish, or preparation of the manuscript.

## Conflict of Interest

The authors declare that the research was conducted in the absence of any commercial or financial relationships that could be construed as a potential conflict of interest.

## Publisher's Note

All claims expressed in this article are solely those of the authors and do not necessarily represent those of their affiliated organizations, or those of the publisher, the editors and the reviewers. Any product that may be evaluated in this article, or claim that may be made by its manufacturer, is not guaranteed or endorsed by the publisher.
